# Reemergence of Intravenous Drug Use as Risk Factor for Candidemia, Massachusetts, USA 

**DOI:** 10.3201/eid2404.171807

**Published:** 2018-04

**Authors:** Nongnooch Poowanawittayakom, Anamika Dutta, Shannon Stock, Sunkaru Touray, Richard T. Ellison, Stuart M. Levitz

**Affiliations:** University of Massachusetts Medical School, Worcester, Massachusetts, USA (N. Poowanawittayakom, S. Touray, R.T. Ellison III, S.M. Levitz);; College of the Holy Cross, Worcester (A. Dutta, S. Stock)

**Keywords:** candidemia, candidiasis, Candida albicans, intravenous substance abuse, heroin, endocarditis, fungi, fungal infections, Massachusetts, United States

## Abstract

The epidemic of illicit intravenous drug use (IVDU) in the United States has been accompanied by a surge in drug overdose deaths and infectious sequelae. *Candida albicans* infections were associated with injection of contaminated impure brown heroin in the 1970s–1990s; however, candidiasis accompanying IVDU became considerably rarer as the purity of the heroin supply increased. We reviewed cases of candidemia occurring over a recent 7-year period in persons >14 years of age at a tertiary care hospital in central Massachusetts. Of the 198 patients with candidemia, 24 cases occurred in patients with a history of IVDU. Compared with non-IVDU patients, those with a history of IVDU were more likely to have non-albicans *Candida*, be co-infected with hepatitis C, and have end-organ involvement, including endocarditis and osteomyelitis. Thus, IVDU appears to be reemerging as a risk factor for invasive candidiasis.

## Abstract 2

Drug users were more likely to have non-albicans *Candida*, be co-infected with hepatitis C, and have end-organ involvement.

Invasive fungal infections resulting from candidiasis are notable causes of illness and death in both adults and pediatric patients (*1*–*4*). *C. albicans* accounts for approximately half the cases of candidemia. For example, in a study of 2,019 candidemia cases during 2004–2008, *C. albicans* was responsible for 46% of the cases, followed by *C. glabrata* (26%), *C. parapsilosis* (16%), *C. tropicalis* (8%), and *C. krusei* (3%) (*1*). Risk factors for invasive *Candida* infections include immunocompromised status, central venous catheters, broad-spectrum antimicrobial drugs, kidney disease requiring dialysis, and genitourinary and gastrointestinal surgery or procedures (*5*).

Opioid addiction has emerged as an increasingly crucial public health problem in the United States. More than 50,000 persons died from drug overdoses in the United States in 2015, an increase of >500% since 1990 (*6*). Massachusetts has been one of the states hardest hit by this epidemic; an estimated 2,190 opioid-related deaths occurred in 2016, which is a ≈4-fold increase since 2010, when 560 such deaths occurred (*7*). In addition to deaths from overdoses, infectious sequelae from intravenous drug use (IVDU) commonly occur, including acute infections such as abscesses at injection sites and endocarditis and chronic infections such as HIV and hepatitis C.

During the 1970s–1990s, outbreaks were reported of *C. albicans* infections in intravenous drug users who used impure brown heroin (*8*,*9*). The source of the *Candida* was thought to be contaminated lemon juices used to dissolve the heroin (*10*–*12*). Clinical manifestations in the affected patients included nodular and pustular cutaneous lesions, chorioretinitis, and osteoarticular infection (*8*,*9*,*11*). After the 1990s, the general purity of heroin supplies increased, negating the need to use acidic sources such as lemon juice as solvents; thus, the problem of candidiasis associated with IVDU mostly disappeared. However, rare case reports of candidiasis syndromes associated with IVDU, such as endophthalmitis associated with injecting contaminated buprenorphine, continued to appear in the literature (*13*,*14*).

Recently, we noticed an apparent increase in cases of invasive candidiasis associated with IVDU at UMassMemorial Medical Center, Worcester, Massachusetts, USA. We therefore retrospectively reviewed all cases of candidemia seen at the hospital over a 7-year period. We compared cases in patients with a history of IVDU with those without such a history with regard to demographic, epidemiologic, clinical, laboratory, and microbiological features.

## Methods

UMassMemorial Medical Center is a 781-bed academic medical center affiliated with the University of Massachusetts Medical School and located in central Massachusetts. The study was approved by the University of Massachusetts Medical School (UMMS) Institutional Review Board (IRB). Patients >14 years of age with a positive blood culture for *Candida* species during January 1, 2010–January 31, 2017, were included in the study. We excluded patients <14 years of age, as they were felt to be unlikely to have a history of IVDU. The protocol approved by the IRB also excluded the study of prisoners; however, none of the patients was excluded for this reason.

We identified cases of candidemia by a query of the hospital electronic medical records (EMRs) using Theradoc (Premier Inc., Charlotte, NC, USA). We then reviewed the EMRs of the patients with candidemia for any history of IVDU; we classified cases as IVDU and non-IVDU on the basis of this chart review. We included cases in the IVDU group if there was any history of IVDU, even if remote. We reviewed admission notes, infectious diseases consult notes, psychiatry consult notes, discharge notes, and toxicology screens. We then conducted a further review of the EMRs to extract data on the epidemiology, risk factors, clinical manifestations, microbiology, laboratory findings, and outcomes. The IRB protocol forbade contacting the patients directly.

We performed *Candida* speciation using standard techniques, including the Vitek 2-Yeast System, Vitek-MS, and API 20C (bioMérieux, Marcy l’Étoile, France). We compared the variables in the tables between the IVDU and non-IVDU populations using the Fisher exact test for categorical variables and the Wilcoxon rank sum test for continuous variables. These tests are nonparametric and avoid the need for large sample sizes. We defined statistical significance as a p value <0.05.

## Results

### Epidemiologic Data and Risk Factors 

During January 1, 2010–January 31, 2017, a total of 198 patients had positive blood cultures for *Candida* species. Using the criteria noted in the previous section, we categorized 24 (12%) patients as being in the IVDU group and 174 (88%) patients in the non-IVDU group. Salient baseline characteristics of the study population are shown in Table 1. Patients in the IVDU group were significantly younger and more likely to have had a positive test for hepatitis C compared with patients in the non-IVDU group (p<0.001 for both variables). Although the numbers were small, a significantly higher percentage of patients in the IVDU group had prosthetic valves (p = 0.013). All 3 of the patients in the IVDU group with a prosthetic valve had valve replacement because of prior episodes of endocarditis. None of the other baseline characteristics we studied differed significantly between the groups. Only 1 patient had known HIV infection.

**Table 1:** Baseline characteristics and risk factors for candidemia, categorized by IVDU and non-IVDU groups, among patients at a tertiary care hospital, Massachusetts, USA, 2010–2017*

Characteristics	IVDU	Non-IVDU	p value
No. patients	24	174	
Median age (IQR)	43.5 (14.8)	64.0 (19.0)	<0.001
Female sex†	6 (25.0)	70 (40.2)	0.183
Prosthetic valve	3 (12.5)	2 (1.2)	0.013
Hepatitis C	14 (58.3)	14 (8.1)	<0.001
HIV	0	1 (0.6)	1.00
History of malignancy	2 (8.3)	42 (24.1)	0.114
Diabetes	3 (12.5)	55 (31.6)	0.058
Systemic immunosuppression‡	3 (12.5)	28 (16.1)	1.00
Central intravenous line	9 (37.5)	67 (38.5)	1.00
History of broad-spectrum antimicrobial drug exposure§	4 (16.7)	27 (15.5)	1.00
Kidney disease, on dialysis	3 (12.5)	5 (2.9)	0.058
Genitourinary surgery/procedure§	0	14 (8.1)	0.226
Gastrointestinal surgery/procedure§	3 (12.5)	22 (12.6)	1.00

*Values are no. (%) patients except as indicated. IQR, interquartile range; IVDU, intravenous drug use. †In all patients, gender and biological sex were identical. ‡Includes active chemotherapy, systemic corticosteroid use, and immunosuppression within 3 months before the onset of candidemia. § Procedure performed <30 days before onset of candidemia.

We compared the prevalence of many of the established risk factors for candidemia in IVDU and non-IVDU groups (Table 1). Although the differences were not statistically significant, patients in the non-IVDU group were more likely to have diabetes (31.6% v. 12.5%) and a history of malignancy (24.1% vs. 8.3%). We noted no significant differences between the 2 groups with regard to systemic immunosuppression, genitourinary procedures, kidney disease requiring dialysis, central intravenous lines, gastrointestinal surgery procedures, or history of broad-spectrum antimicrobial drug use.

Of the 24 cases of IVDU-associated candidemia recorded during the 7-year period of the study, only 2 occurred during the first 2 years. In contrast, 9 cases occurred in 2016, the last year of the study (Figure). The number of candidemia cases was not associated with diminished IVDU in the last 2 years of the study.

**Figure:**
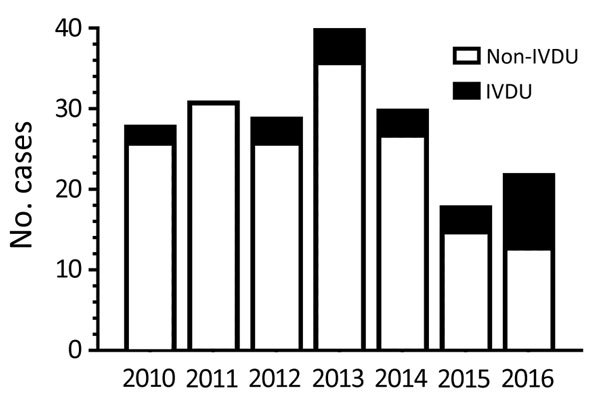
Distribution of candidemia cases associated with IVDU and non-IVDU by year at a tertiary care hospital, Massachusetts, USA, 2010–2017. Candidemia cases were divided into IVDU and non-IVDU groups and then plotted as a function of the year the patient had positive blood cultures for *Candida*. There were no positive blood cultures in January 2017, the last month of the study.

### Features of Patients with Candidemia and History of IVDU

Details about the patients with a history of IVDU are provided in Table 2. Most of the patients gave a history of use of heroin, cocaine, or both. However, prescription opioids were also commonly found when toxicology screens were obtained.

**Table 2:** Summary of characteristics of patients with candidemia and a history of IVDU admitted to a tertiary care hospital, Massachusetts, USA, 2010–2017*

Pt no.	Age, y/ sex	Date admitted	IVDU, by history	Toxicology screen	Last use, by history	*Candida* species	Vegetations on echo	Antifungal therapy (duration)	Outcome
1	32/M	2010 Jan	Heroin	Heroin, morphine, cocaine	5 d	*C. parapsilosis*	No	Fluc (14 d)	Cured
2	28/M	2010 Oct	Heroin	ND	6 mo	*C. parapsilosis*	No	Fluc (14 d)	Cured
3	52/F	2012 Jul	Heroin, cocaine	Opiates	NA	*C. glabrata*	No	MF (8 d) then fluc (20 d)	Cured
4	43/F	2012 Oct	Heroin	Cocaine, opiates†	1.5 d	*C. parapsilosis*	Yes (aortic, mitral)	None	Died
5	59/M	2012 Nov	Not documented	ND	30 y	*C. glabrata*	No	Fluc (14 d)	Died
6	54/M	2013 Mar	Heroin, cocaine	Methadone, opiates	2 y	*C. parapsilosis*	No	MF (3 d) then fluc (25 d)	Cured
7	43/F	2013 Apr	Heroin	ND	NA	*C. albicans*	No	MF (3 d) then fluc (25 d)	Cured
8	31/M	2013 Aug	Heroin	Opiates	2 y	*C. albicans*	No	MF (2 d) then fluc (12 d)	Cured
9	36/M	2013 Dec	Heroin	Oxycodone, hydromorphone	6 d	*C. glabrata,* *C. parapsilosis*	No	None	Lost to follow up
10	49 /F	2014 Jan	Unspecified narcotic	Opiates	NA	*C. glabrata*	No	MF (3 d)	Died
11	38/F	2014 Feb	Heroin	Oxycodone, oxymorphone	2 y	*C. albicans*	Possible (tricuspid on TTE, TEE negative)	Fluc (2 d) then MF (40 d)	Remission (had epidural abscess)
12	50/M	2014 Aug	Heroin	Morphine	6 mo	*C. tropicalis*	No	Fluc (14 d)	Cured
13	49/M	2015 Mar	Cocaine	ND	Unknown	*C. lipolytica*	No	Fluc (14 d)	Cured
14	22/M	2015 Jun	Heroin	Marijuana	Day admitted	*C. glabrata*	No	MF (3 d) then fluc (25 d)	Cured
15	53/M	2015 Sep	Cocaine	Cocaine	Remote	*C. tropicalis*	No	Fluc (2 d)	Died
16	31/F	2016 Jan	Cocaine	Morphine	NA	*C. tropicalis*	No	Fluc (14d)	Cured
17	37/M	2016 Feb	Heroin	ND	3 wk	*C. glabrata*	No	Fluc (14d)	Cured
18	35/M	2016 Mar	Heroin, Cocaine	Cocaine, opiates†	NA	*C. albicans*	No	MF (4 d) then fluc (24 d)	Cured
19	44/M	2016 Apr	Cocaine, heroin	Buprenorphine, norbuprenorphine	7 mo	*C. parapsilosis*	Yes (aortic)	Fluc (indefinite)	Remission follow up TTE after 3 mo: no vegetation
20	47/M	2016 Apr	Cocaine	ND	1 y	*C. albicans*	No	MF (6 d) then fluc (8d)	Cured
21	32/F	2016 May	Heroin	Methadone, oxycodone	2 wk	*C. albicans*	Yes (mitral)	AmpB (1 d) then fluc and vori (indefinite)‡	Remission follow up TTE after 3 mo: no vegetation
22	46/M	2016 May	Cocaine, heroin	ND	Day admitted	*C. glabrata*	No	Fluc (21 d)	Cured
23	40/M	2016 Jun	Heroin	Opiates	Day admitted	*C. albicans,* *C. glabrata*	No	MF (13 d) then fluc (1 d)	Cured
24	54/M	2016 Sep	Heroin	ND	1 mo	*C. parapsilosis*	Yes (aortic)	MF (5 d) then ampB and 5FC (5 d)	Died

*ampB, amphotericin B; 5FC, 5-flucytosine; echo, echocardiogram; fluc, fluconazole; IVDU, intravenous drug use; MF, micafungin; NA, not available; ND, not done; Pt, patient; TEE, transesophageal echocardiogram; TTE, transthoracic echocardiogram; vori, voriconazole. †Positive toxicology screen was obtained during a previous admission. ‡Patient developed hypercalcemia after courses of fluconazole and treatment was changed to voriconazole.

### Organ System Involvement

We determined the prevalence of brain abscesses, osteomyelitis, retinitis, skin lesions, septic emboli to the lungs, and endocarditis (Table 3). Although endocarditis and osteomyelitis were seen infrequently, the IVDU group had a significantly higher percentage of cases of these 2 diseases compared with the non-IVDU group.

**Table 3:** Organ system involvement in patients with candidemia at a tertiary care hospital, Massachusetts, USA, 2010–2017*

Manifestation	No. (%) patients		p value
	IVDU, n = 24	Non-IVDU, n = 174	
Endocarditis	4 (16.7)	5 (2.9)	0.014
Brain abscess	1 (4.2)	0	0.121
Septic emboli, lung	1 (4.2)	0	0.121
Retinitis	0	4 (2.3)	1.00
Osteomyelitis	2 (8.3)	0	0.014

*IVDU, intravenous drug use.

### *Candida* Species

*C. albicans* was isolated from the bloodstream in a significantly higher percentage of non-IVDU patients compared with IVDU patients (Table 4). In contrast, the percentage of *Candida* isolates identified as *C. parapsilosis* was significantly higher in the IVDU group. Other *Candida* species were isolated, mostly commonly *C. glabrata* and *C. tropicalis*, but their distribution was not significantly different between the IVDU and non-IVDU groups. Two patients in each group were co-infected with 2 *Candida* species.

**Table 4:** *Candida* species isolated from blood of patients with candidemia at a tertiary care hospital, Massachusetts, USA, 2010–2017*

Organisms	No. (%) patients		p value
	IVDU, n = 24	Non-IVDU, n = 174	
*C. albicans*	7 (29.2)†	93 (53.5)	0.03
*C. glabrata*	8 (33.3)	39 (22.4)	0.304
*C. parapsilosis*	8 (33.3)	25 (14.4)	0.036
*C. tropicalis*	3 (12.5)	13 (7.5)	0.419
*C. dubliniensis*	0	2 (1.2)	1.00
*C. guilliermondii*	0	1 (0.6)	1.00
*C. krusei*	0	1 (0.6)	1.00
*C. lipolytica*	0	1 (0.6)	1.00
*C. kefyr*	0	1 (0.6)	1.00
Co-infected	2 (8.3)	2 (1.2)	0.073

*IVDU, intravenous drug use. †Percentages do not add up to 100% because patients co-infected with 2 species are counted twice.

### Outcome Data

Mortality rates and length of hospitalization were lower in the IVDU group compared with the non-IVDU group (Table 5). However, the differences were not statistically significant.

**Table 5:** Outcomes of patients with candidemia, Massachusetts, USA, 2010–2017*

Outcome	IVDU, n = 24	Non-IVDU, n = 174	p value
Death, no. (%) patients	5 (20.8)	60 (34.5)	0.247
Median length of hospital stay (IQR), d	11 (17.5)	19 (27.5)	0.059

*IQR, interquartile range; IVDU, intravenous drug use.

## Discussion

Our data establish that, in the setting of the ongoing opioid epidemic, candidemia appears to have emerged as a serious clinical problem in the IVDU population in central Massachusetts. Major differences appear, however, between the cases reported in the literature that were associated with injection of brown heroin and the ones in this case series. Cutaneous, ocular, or osteoarticular involvement were commonly observed in the brown heroin users with candidiasis (*8*,*9*) but were rarely seen in the IVDU case-patients we report. In contrast, although all the cases in our study featured candidemia by definition, blood cultures were rarely positive for candidemia in the brown heroin cases.

As discussed earlier, in the brown heroin cases, the infections were linked to *C. albicans* contamination of lemon juice used to solubilize the heroin; blood cultures, when positive, grew only *C. albicans* (*8*,*9*). In our case series, *C. albicans* was the third most common species, behind *C. glabrata* and *C. parapsilosis*. The finding of different species of *Candida* argues against a common source of infection. Rather, endogenous sources of *Candida* seem likely, because this fungus can colonize the skin and mouth; injection drug users may lick their needles before insertion into the skin. However, our IRB protocol did not permit us to interview subjects regarding injection habits or to culture their drugs and injection paraphernalia. Thus, the source of the *Candida* species isolated from the patients is speculative, and future studies are needed to address this question.

*C. glabrata* and *C. parapsilosis* are typically thought of as nosocomial pathogens, with spread of the pathogen facilitated by contaminated inanimate surfaces and person-to-person contact (*15*,*16*). However, both species can also be gut colonizers and commensals of human skin (*16*). *C. glabrata* is often resistant to fluconazole; a risk factor for infection with this species is prior receipt of fluconazole (*15*). However, none of our patients was known to have received fluconazole before contracting candidemia.

In our study, we found that the candidemia patients in the IVDU group were younger and more likely to be co-infected with hepatitis C than were patients in the non-IVDU group. This finding likely reflects the relatively young age of the IVDU population and the high prevalence of hepatitis C infection for this group (*17*). Even though more than half of the patients in the IVDU group were co-infected with hepatitis C, no patient had HIV infection. This finding is consistent with a 2012 report documenting declining rates of HIV infection and increasing rates of hepatitis C among injection drug users in Massachusetts (*18*).

In a recent multicenter study of >2,000 patients with candidemia, *C. albicans* was responsible for 46% of the cases, followed by *C. glabrata* (26%), *C. parapsilosis* (16%), *C. tropicalis* (8%), and *C. krusei* (3%) (*1*). In our study, the species distribution in the non-IVDU population with candidemia was very similar, with slightly more than half the cases attributable to *C. albicans* (Table 4). In contrast, only 7 (29%) of 24 candidemia cases in the IVDU population were caused by *C. albicans*, and 1 of those cases was a co-infection with *C. glabrata*. Perhaps reflecting these patients’ younger age and fewer concurrent conditions, the mortality rate was lower for the IVDU population compared with the non-IVDU population, although the difference was not statistically significant. Nevertheless, 5 (21%) of 24 patients in the IVDU group died, emphasizing that candidemia in the IVDU population is a serious disease associated with a serious risk of death.

Our retrospective study does not shed light on the optimal therapy for IVDU-associated candidemia. However, it seems reasonable to begin to treat patients with an echinocandin, according to the Infectious Diseases Society of America clinical practice guideline for the treatment of candidemia (*19*), pending identification and susceptibility testing of the isolate. One third of our IVDU-associated candidemia cases were caused by *C. glabrata* which, as noted earlier, is often resistant to fluconazole (*15*). In addition, the superiority of an echinocandin over fluconazole was demonstrated in a multicenter trial of patients with candidemia (*20*). However, a disadvantage of the echinocandins is their need to be given intravenously, a particular problem in the patient population with IVDU, because they may use intravenous lines for drug injection. In clinically stable patients with susceptible isolates and without endocarditis, transition to fluconazole can be considered when the patient is ready for discharge.

Several limitations should be taken into account when interpreting the data we present. The study was retrospective, and because of our IRB protocol, we were not permitted to contact the patients directly to verify their histories or obtain additional information. Moreover, it is often difficult to obtain an accurate history of IVDU from patients. Thus, because it was not known whether the injection drug use was current or not, we cannot be certain that the IVDU contributed to the risk of developing candidemia. Indeed, it is possible that some of the patients were misclassified and there may be inaccuracies in variables such as when the patients last injected drugs and what drugs were injected. For this reason, when designing the study, we prospectively decided to include all patients with a history of IVDU, however remote, in the IVDU group. Second, although it is very likely that the trend toward increasing numbers of IVDU-associated candidemia is real, it is possible that the way IVDU is recorded has changed over the years because healthcare providers are now more attuned to the opioid epidemic and thus record the history of IVDU more often than they did in the past. Third, although the number of cases was relatively large, the study was not powered to detect differences in uncommon events between the IVDU and non-IVDU groups. In addition, multivariate analyses were not performed, so some statistically significant associations may be confounded. Fourth, there may have been a bias toward ordering certain tests, such as hepatitis C serology and echocardiography, in the IVDU-associated candidemia group. Finally, our study was a single-center study from a tertiary care institution. It remains to be seen whether candidemia in IVDU is mostly a local phenomenon peculiar to central Massachusetts or it is emerging at other medical centers in other geographic locations.

These limitations notwithstanding, our findings establish candidemia as an underappreciated outcome of the opioid epidemic gripping the United States. Moreover, we observed a marked increase in the number of cases during the last year of the study. Although this finding may reflect random variations, it is of concern that the problem may be worsening. In Worcester County, where UMassMemorial Hospital is located, the number of opioid-related overdose deaths increased every year during the 7-year study period, from 80 in 2010 to 251 in 2016 (*7*). Although many of the press reports surrounding illicit opioid use have centered on deaths from overdose, infections remain a serious cause of illness and death in persons with IVDU. Invasive candidiasis must be considered in the differential diagnosis of patients with infectious sequelae of IVDU.
